# Clinical and Genetic Features in Knobloch Syndrome: A Case Series Reporting Two Previously Unreported *COL18A1* Variants

**DOI:** 10.3390/children13081014

**Published:** 2026-07-30

**Authors:** Omar Anwar Sadat, Michael Shi, Haseeb Mahmud, Geoffrey Bradford, James Vernon Odom, Monique Leys

**Affiliations:** Department of Ophthalmology and Visual Sciences, West Virginia University, Morgantown, WV 26506, USA; omar.sadat@hsc.wvu.edu (O.A.S.); michael.shi@hsc.wvu.edu (M.S.); haseeb.mahmud@hsc.wvu.edu (H.M.); bradfordg@hsc.wvu.edu (G.B.); jodom@hsc.wvu.edu (J.V.O.)

**Keywords:** Knobloch syndrome, *COL18A1*, inherited retinal disease, macular atrophy, vitreoretinal degeneration, novel variant

## Abstract

**Purpose:** Knobloch syndrome (KS) is a rare autosomal recessive disorder caused by biallelic variants in *COL18A1* and characterized by high myopia, vitreoretinal degeneration, and occipital defects. This study examines genotype–phenotype correlations in patients with KS and reports two previously unreported *COL18A1* variants. **Methods:** Retrospective case series from January 2010 to December 2025. From a cohort of 627 patients with inherited retinal diseases (IRDs) seen at the West Virginia University Eye Institute (WVU), all patients with clinical features of KS and pathogenic, likely pathogenic, or candidate *COL18A1* variants were identified. Clinical records, multimodal retinal imaging, and electrodiagnostic testing results were reviewed and compared. **Results:** Four patients with clinical features of KS and genetic variants classified as pathogenic, likely pathogenic, or variants of uncertain significance in *COL18A1* were identified, representing 0.64% of this single-center IRD cohort at WVU. All patients demonstrated the core ophthalmic features of KS: nystagmus, high myopia, macular atrophy, and vitreous degeneration. Systemic manifestations included seizure disorder, aplasia cutis congenita, renal anomalies, occipital encephalocele, and global developmental delay. Four distinct *COL18A1* variants were identified, including two previously unreported variants: c.2673dupC p.Gly892Argfs*9 and c.3827C > T p.Ser1276Leu (variant of uncertain significance). **Conclusions:** We describe two previously unreported *COL18A1* variants in patients with clinical features of Knobloch syndrome. This series reinforces the consistent ophthalmic phenotype of KS and highlights phenotypic variability in systemic features. Genetic testing is essential for definitive diagnosis, and KS should be considered in any child presenting with early onset nystagmus, high myopia, macular atrophy, and profoundly abnormal electroretinography.

## 1. Introduction

Knobloch syndrome (KS; OMIM #267750) is a rare autosomal recessive disorder first described by Knobloch and Layer in 1971 in a family with high myopia, vitreoretinal degeneration, retinal detachment, and occipital encephalocele [1]. Since the initial report, the phenotypic spectrum of KS has expanded considerably, with fewer than 100 cases documented in the literature to date.

The genetic basis of KS was established in 2000 by Sertié et al., who identified loss-of-function mutations in *COL18A1* (OMIM *120328), located on chromosome 21q22.3, as the causative molecular defect [2]. *COL18A1* encodes the alpha-1 chain of type XVIII collagen, a heparan sulfate proteoglycan component of basement membranes throughout the body. In the eye, type XVIII collagen is highly expressed in the vitreous, iris, retina, and retinal pigment epithelium, where it plays a critical role in anchoring the vitreous fibrils to the inner limiting membrane and in regulating retinal vasculature development [2,3]. Loss of functional type XVIII collagen leads to structural instability of the vitreoretinal interface, predisposing to the characteristic ophthalmic findings of KS.

The classic clinical triad of KS consists of: (1) high myopia and vitreoretinal degeneration, (2) macular abnormalities including atrophy or coloboma, and (3) midline occipital defects ranging from aplasia cutis congenita to frank encephalocele [4,5]. However, KS demonstrates marked phenotypic heterogeneity; occipital defects may be absent or subtle, and extraocular manifestations including renal anomalies, seizures, and developmental delay have been described [6,7]. Electroretinography (ERG) in KS typically reveals a cone–rod pattern of dysfunction, though ERG findings have not been uniformly characterized across reported cases [4,5,8].

To date, over 60 distinct *COL18A1* pathogenic variants have been reported, including nonsense, frameshift, missense, and splice site variants [5,9]. Despite this growing catalog, the genotype–phenotype correlations in KS remain incompletely understood. Here, we present four patients with clinical features of KS and genetic variants classified as pathogenic, likely pathogenic, or variants of uncertain significance (VUS) in *COL18A1* from a cohort of 627 patients with inherited retinal disease, including the identification of two previously unreported *COL18A1* variants, and describe their clinical and multimodal imaging features.

## 2. Materials and Methods

This study was a retrospective case series conducted at the West Virginia University Eye Institute, reviewing clinical records from January 2010 through December 2025. The study adhered to the tenets of the Declaration of Helsinki. Written consent for publication of clinical images and case presentation was obtained for each patient presented. The study was reviewed by the West Virginia University Institutional Review Board and determined to be exempt from IRB review under Category 4 (Secondary Research on Data or Specimens) (IRB ID: STUDY00000533; determination dated 1 July 2026). Data collection was conducted from May 2025 to July 2026.

We reviewed the records of 627 consecutive patients who presented with clinically suspected or genetically confirmed inherited retinal disease and who underwent next-generation sequencing (NGS) gene panel testing or whole-exome sequencing (WES). Patients were drawn from two clinical sources at West Virginia University: the Inherited Retinal Disease clinic at the WVU Eye Institute and the WVU Department of Genetics. Genetic testing across this cohort was performed on a range of platforms that evolved over the study period, including institution- and program-specific NGS panels (the Spark Therapeutics ID YOUR IRD program, performed sequentially at Prevention Genetics [39 genes], then an RP65-only panel, and then Invitae [248, 293, and 330 genes]; the Foundation Fighting Blindness My Retina Tracker program, performed at Blueprint Genetics [333 genes plus the mitochondrial genome], and later, Prevention Genetics [110 genes plus the mitochondrial genome]; a Phosphorus 144-gene panel; the University of Iowa Carver Lab panel for selected clinically suspected disorders; and the NEI eyeGENE program), as well as Invitae panel testing and WES performed at GeneDx through the Department of Genetics. Patients were included in this analysis if they had a clinical phenotype consistent with KS and harbored two pathogenic variants, likely pathogenic variants, or VUS in *COL18A1*, as defined by the American College of Medical Genetics and Genomics (ACMG) and Association for Molecular Pathology (AMP) variant interpretation guidelines [10].

Genetic diagnosis in the four patients described here was established using one of the following platforms: the Invitae Inherited Retinal Disorders NGS panel (San Francisco, CA, USA) (330 genes), or WES performed at GeneDx (Gaithersburg, MD, USA). Variant classification followed ACMG/AMP criteria [10]. Variants designated as variants of uncertain significance (VUS) were classified using the same framework and are reported as such. *COL18A1* coverage was not uniform across the platforms and panel versions used in the broader 627-patient cohort; in particular, the Phosphorus 144-gene panel and earlier, smaller iterations of the Invitae panel did not include *COL18A1*. A systematic re-review of genetic testing results across all 627 patients was not feasible, so we cannot exclude the possibility that additional patients with KS-related phenotypes were not identified because of this non-uniform *COL18A1* coverage, particularly prior to 2020.

Clinical data collected for each patient included: best-corrected visual acuity (BCVA), cycloplegic refraction, slit-lamp biomicroscopy findings, fundus examination, and documented systemic features. Multimodal retinal imaging including spectral-domain optical coherence tomography (SD-OCT) (Heidelberg Engineering, Heidelberg, Germany), color fundus photography, and fundus autofluorescence (FAF) (Optos, Dunfermline, Scotland) imaging was reviewed where available. Goldmann kinetic perimetry (Haag Streit, Köniz, Switzerland) was performed where patient age and cooperation permitted. Electroretinography (ERG) was performed when possible given patient age and cooperation. Patient 1 was tested binocularly with jet electrodes using the UTAS cupola (LKC Technologies, Germantown, MD, USA) using the ISCEV 2008 protocol [11]. Patient 4 was tested at age 1 during examination under anesthesia (EUA) using the following modified protocol. After induction of anesthesia, the left eye was patched. Then, the right eye was tested with the RETeval (LKC Technologies, Germantown, MD, USA) using a jet electrode contact lens under standard operating room lights with photopic flash 3.0 cd·s/m^2^ and an average of 5 flashes and 28.3 Hz flicker. Following testing of the right eye, room lights and ambient lights were dimmed as much as possible, and after 20 min of dark adaptation and placement of the jet contact lens electrode in the left eye, a single flash at 3.00 cd·s/m^2^ was used to test the mixed cone–rod response. This non-standard protocol is designed to screen for severe bilateral retinal degeneration during an EUA.

At age 4, the handheld RETeval stimulator (LKC Technologies, Germantown, MD, USA) with sensor strip electrodes was employed. The RETeval is a monocular device, so the two eyes were tested sequentially in the clinic using the ISCEV 2015 protocol [12].

## 3. Results

Four patients with clinical features of KS and pathogenic, likely pathogenic, or candidate *COL18A1* variants were identified from the 627-patient IRD cohort, representing 0.64% of this single-center IRD cohort. Clinical and genetic data are summarized in Table 1. All four patients demonstrated the core ophthalmic features of KS: nystagmus, high myopia, macular atrophy, and vitreous degeneration. Four distinct *COL18A1* variants were identified; two of these—c.2673dupC p.Gly892Argfs*9 and c.3827C > T p.Ser1276Leu—have not been previously described in the literature. Detailed variant-level annotation, including reference transcript, genomic coordinates, zygosity, inheritance/phase where available, gnomAD population frequency, ClinVar status, predicted consequence, and classification date are provided in Table 2.

### 3.1. Patient 1

**Figure 1:**
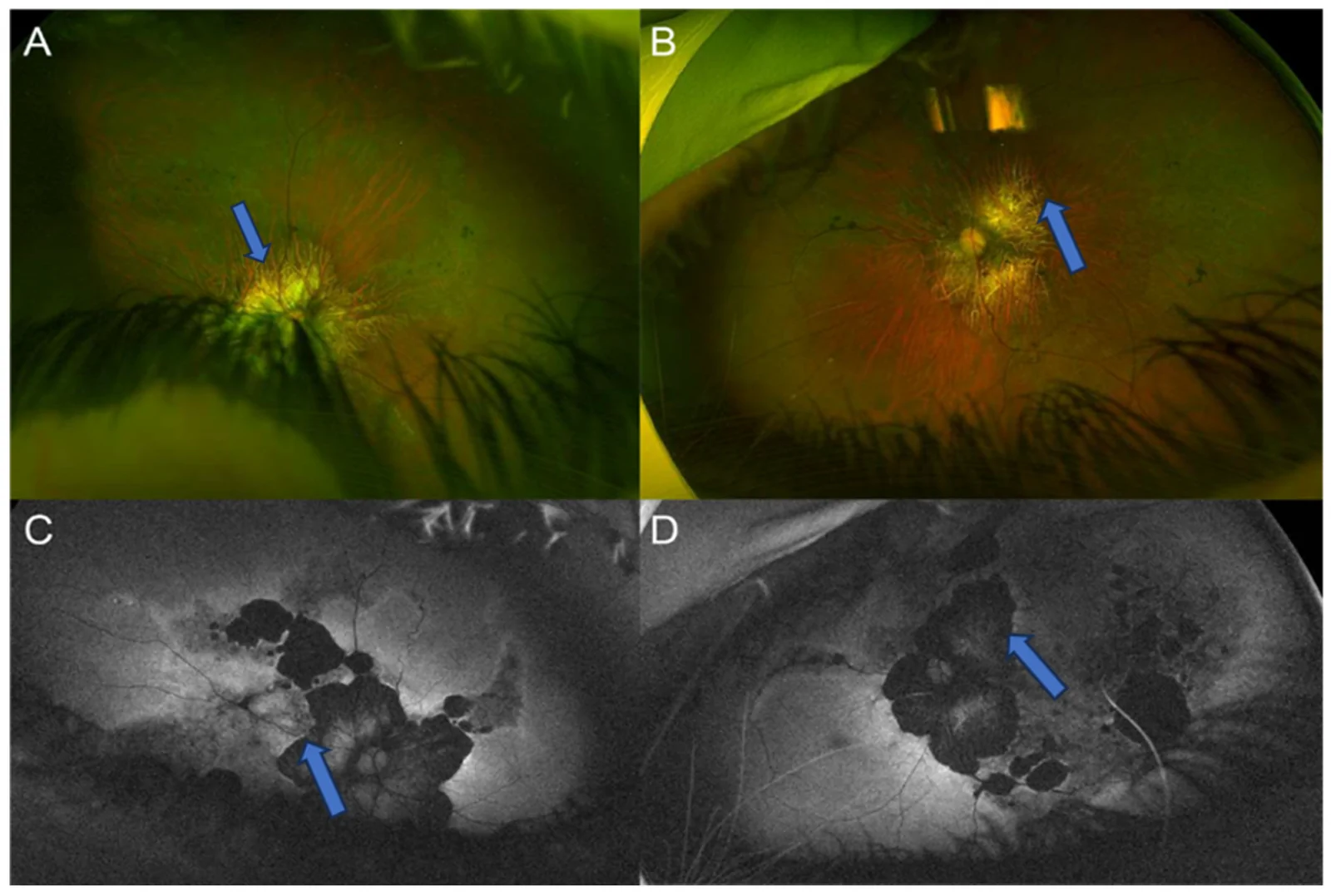
Patient 1. Optos ultra-widefield color fundus photos (**A**,**B**) and fundus autofluorescence photos (**C**,**D**) of the right (**A**,**C**) and left (**B**,**D**) eye showing severe myopic macular atrophy with reduced peripapillary autofluorescence (blue arrows).

A 31-year-old female with a known seizure disorder presented for genetic evaluation of progressive visual impairment. Her early ocular history was notable for nystagmus and high myopia detected at 6 months of age. Refraction revealed severe myopia of −13.00 D OD and −14.00 D OS. Fundus examination showed diffuse macular atrophy, vitreous syneresis, and myopic fundus features bilaterally (Figure 1).

**Figure 2:**
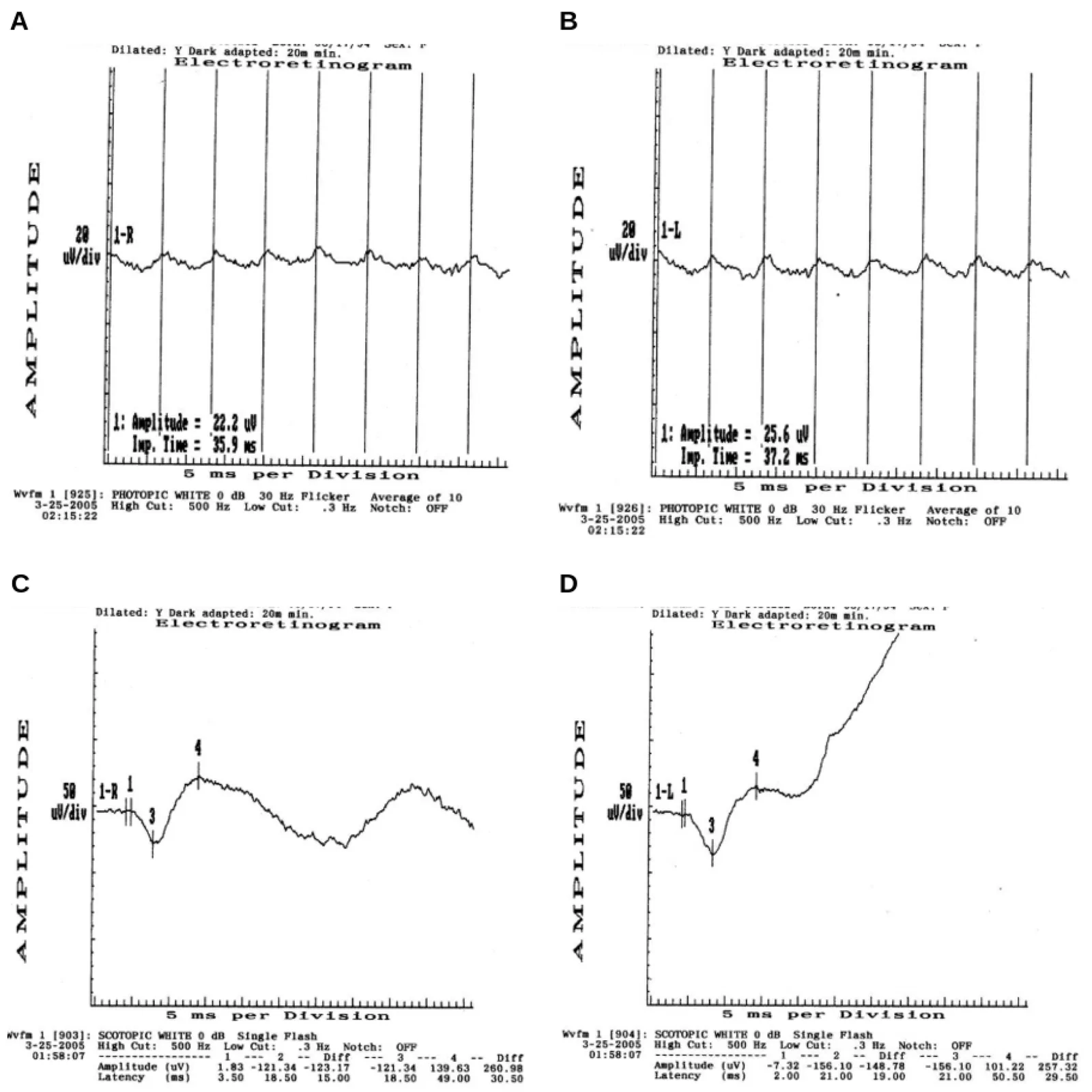
Patient 1. (**A**,**B**) Representative photopic 30 Hz flicker ERG waveforms for the right eye (**A**) and left eye (**B**), showing reduced and delayed flicker amplitude (OD 22.2 µV/35.9 ms; OS 25.6 µV/37.2 ms) relative to normal (60.2 µV/30.5 ms). (**C**,**D**) Representative scotopic (dark-adapted) single-flash combined rod–cone ERG waveforms for the right eye (**C**) and left eye (**D**). The a-wave trough (point 3) and b-wave peak (point 4) are marked, yielding b-wave amplitudes (measured trough-to-peak) of 260.98 µV (OD) and 257.32 µV (OS), with corresponding b-wave implicit times (49 ms and 50.5 ms, respectively) that were relatively normal (382.68 µV/53.3 ms).

At 11 years of age, a full-field ERG using the LKC Utas instrument with jet electrode contact lens electrodes was performed. The ISCEV protocol was followed, and after pupillary dilation and 20 min of dark adaptation scotopic, responses were recorded. The patient was then light adapted for 10 min for the single-flash and 30 Hz flicker ERG. Results showed reduced amplitudes and prolonged implicit times across all conditions, consistent with generalized retinal dysfunction (Figure 2).

**Figure 3:**
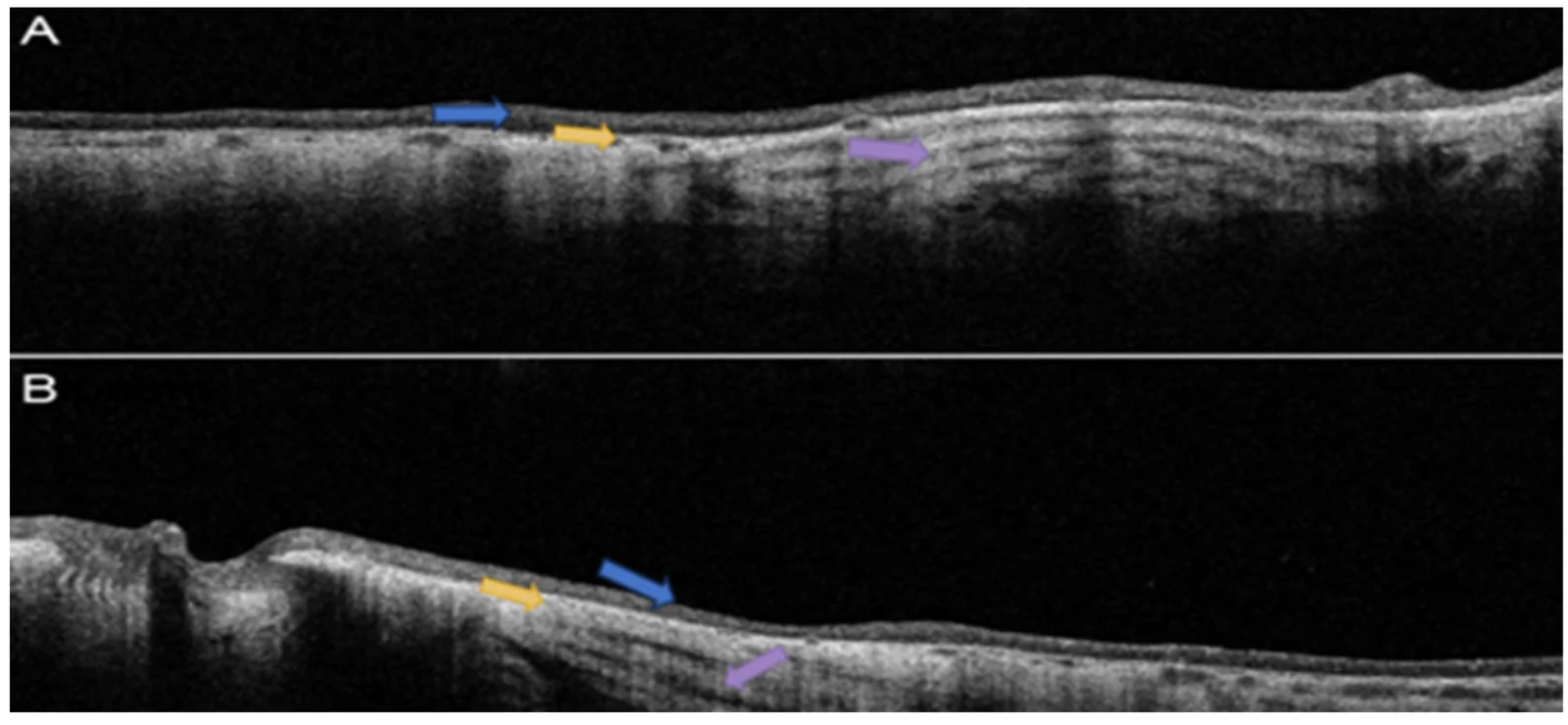
Patient 1. Heidelberg wide-field optical coherence tomography of the macula of the right (**A**) and left (**B**) eye showing severe myopic macular atrophy (blue arrows), choroidal thinning (yellow arrows), and shine-through artifact (purple arrows).

**Figure 4:**
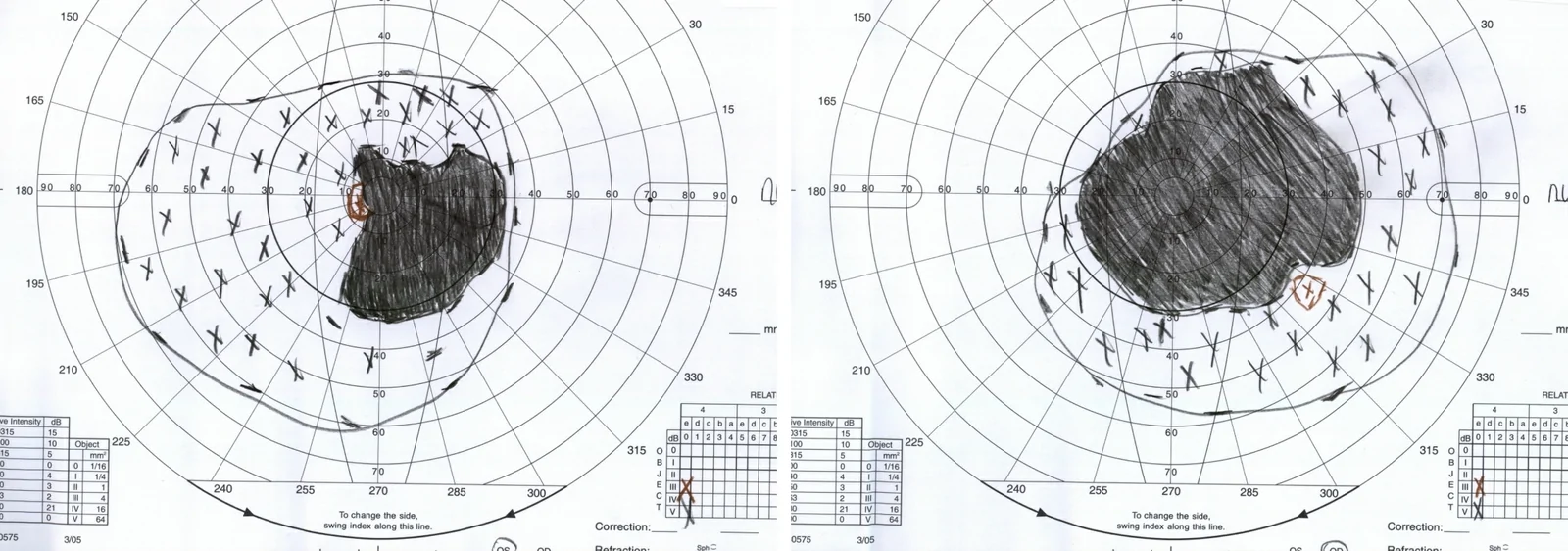
Patient 1. Goldmann visual field showing constriction of visual fields and central scotomas of both eyes correlating to areas of myopic macular atrophy.

**Figure 5:**
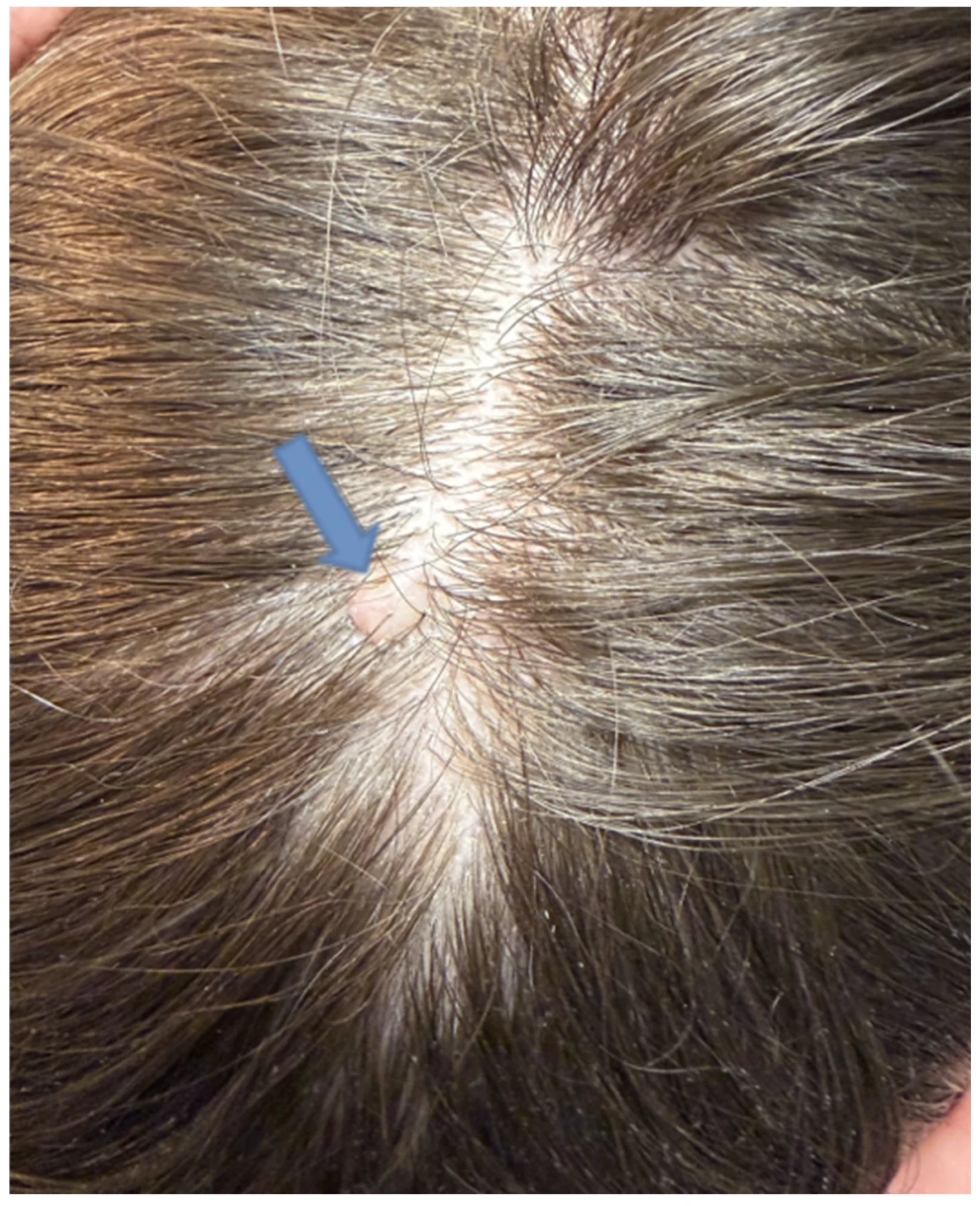
Patient 1. Color photo scalp showing well-circumscribed area of alopecia at the vertex of the scalp with a central atrophic, membranous pink-tan plaque (blue arrow) consistent with aplasia cutis congenita (ACC), a recognized feature within the clinical spectrum of Knobloch syndrome.

At age 13, she developed bilateral posterior subcapsular cataracts and subluxed lenses. She subsequently underwent bilateral cataract extraction and pars plana vitrectomy; postoperative BCVA was 20/400 OU with aphakic contact lens correction (+5.00 OD, +5.50 OS). SD-OCT confirmed severe macular atrophy with loss of the outer nuclear layer and ellipsoid zone (Figure 3). Goldmann visual field testing demonstrated markedly constricted fields bilaterally (Figure 4). Figure 1, Figure 3, Figure 4 and Figure 5 show the patient’s latest exam findings at age 31.

Systemic evaluation revealed she had been suffering from a seizure disorder managed with anticonvulsant therapy. Dermatologic examination demonstrated a well-circumscribed area of alopecia at the vertex of the scalp with a central atrophic, membranous pink-tan plaque consistent with aplasia cutis congenita (ACC), a recognized non-occipital midline manifestation of KS (Figure 5) [13].

The Invitae 330-gene IRD panel identified a homozygous frameshift variant c.3514_3515del p.Leu1172Valfs*72 in *COL18A1*, classified as pathogenic.

### 3.2. Patient 2

Ophthalmology was consulted for evaluation of a 2-day old female born with horizontal nystagmus. Limited bedside examination, including dilated fundoscopy, was suspicious for bilateral optic nerve hypoplasia. MRI of the brain was unremarkable at that time. Systemic evaluation at birth revealed acute kidney injury and increased echogenicity of both kidneys on renal ultrasound that may suggest some architectural irregularity. No occipital scalp defect or encephalocele was identified on physical examination or neuroimaging. After discharge, the patient did not return to our institution until 4 years of age.

**Figure 6:**
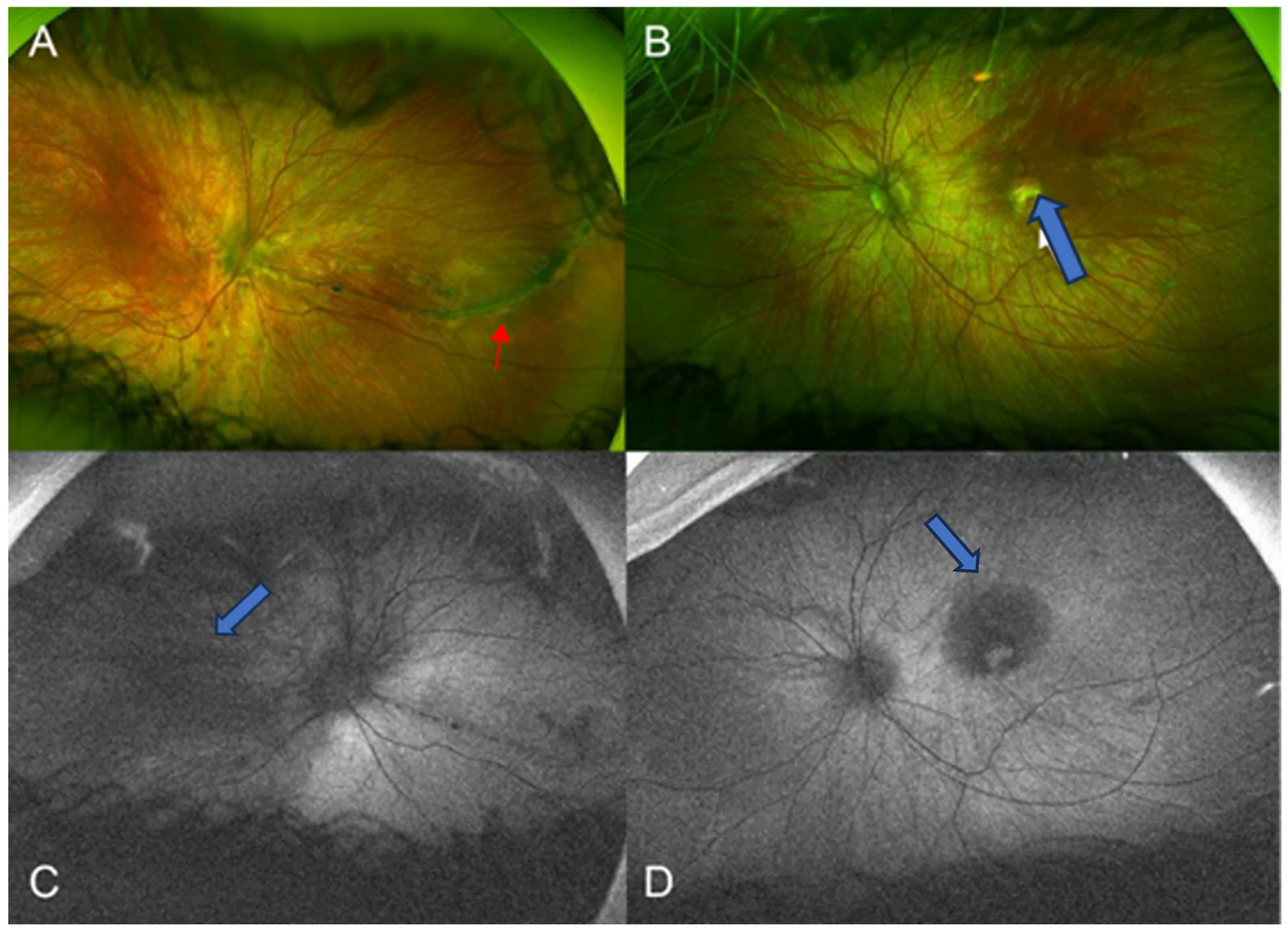
Patient 2. Optos ultra-widefield color fundus photos (**A**,**B**) and fundus autofluorescence photos (**C**,**D**) of the right (**A**,**C**) and left (**B**,**D**) eye showing significant myopic atrophy with reduced autofluorescence (blue arrows), and perivascular lattice (red arrow).

Referring notes revealed she had undergone strabismus surgery for exotropia at 2 years of age. The earliest documented refraction at 4 years of age showed significant anisomyopia (−9.00 D OD and −19.00 D OS), with BCVA of 20/250 OU and an intermittent exotropia of 10 prism diopters in primary gaze. Fundus examination revealed diffuse macular atrophy and vitreous degeneration bilaterally (Figure 6).

By age 6, her cycloplegic refraction remained stable, and her strabismus evolved into a constant right exotropia of 20 prism diopters despite a history of patching and glasses wear.

WES (GeneDx) identified the homozygous frameshift variant c.3514_3515del p.Leu1172Valfs*72 in *COL18A1*, identical to that identified in Patient 1, classified as pathogenic. The presence of the same homozygous variant in two unrelated patients from the same geographic region may suggest shared ancestry/unknown consanguinity or a recurrent variant, consistent with prior reports of recurrent *COL18A1* variants in unrelated families [5].

### 3.3. Patient 3

**Figure 7:**
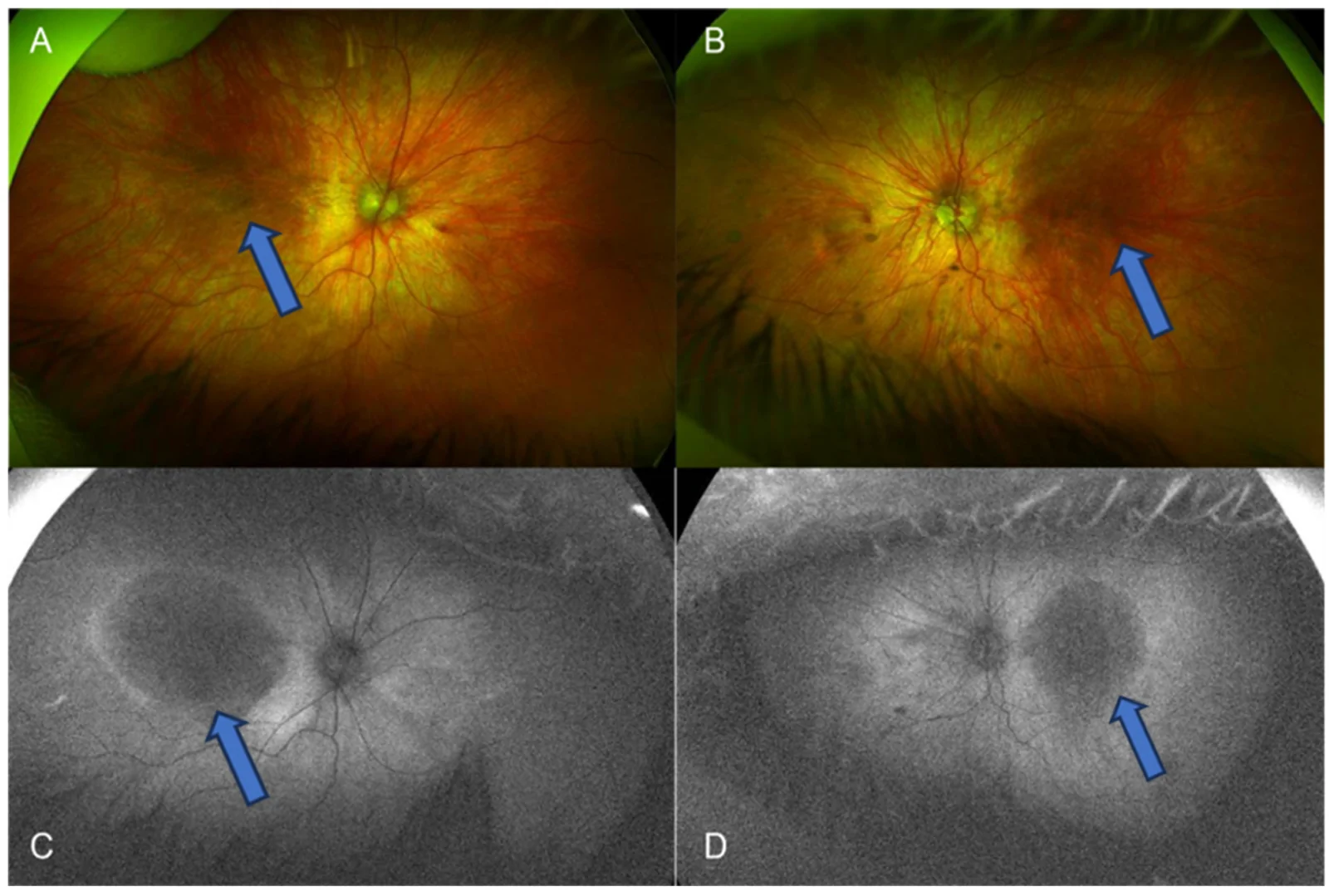
Patient 3. Optos ultra-widefield color fundus photos (**A**,**B**) and fundus autofluorescence photos (**C**,**D**) of the right (**A**,**C**) and left (**B**,**D**) eye showing severe myopic macular atrophy correlating with reduced macular autofluorescence marked (blue arrows).

**Figure 8:**
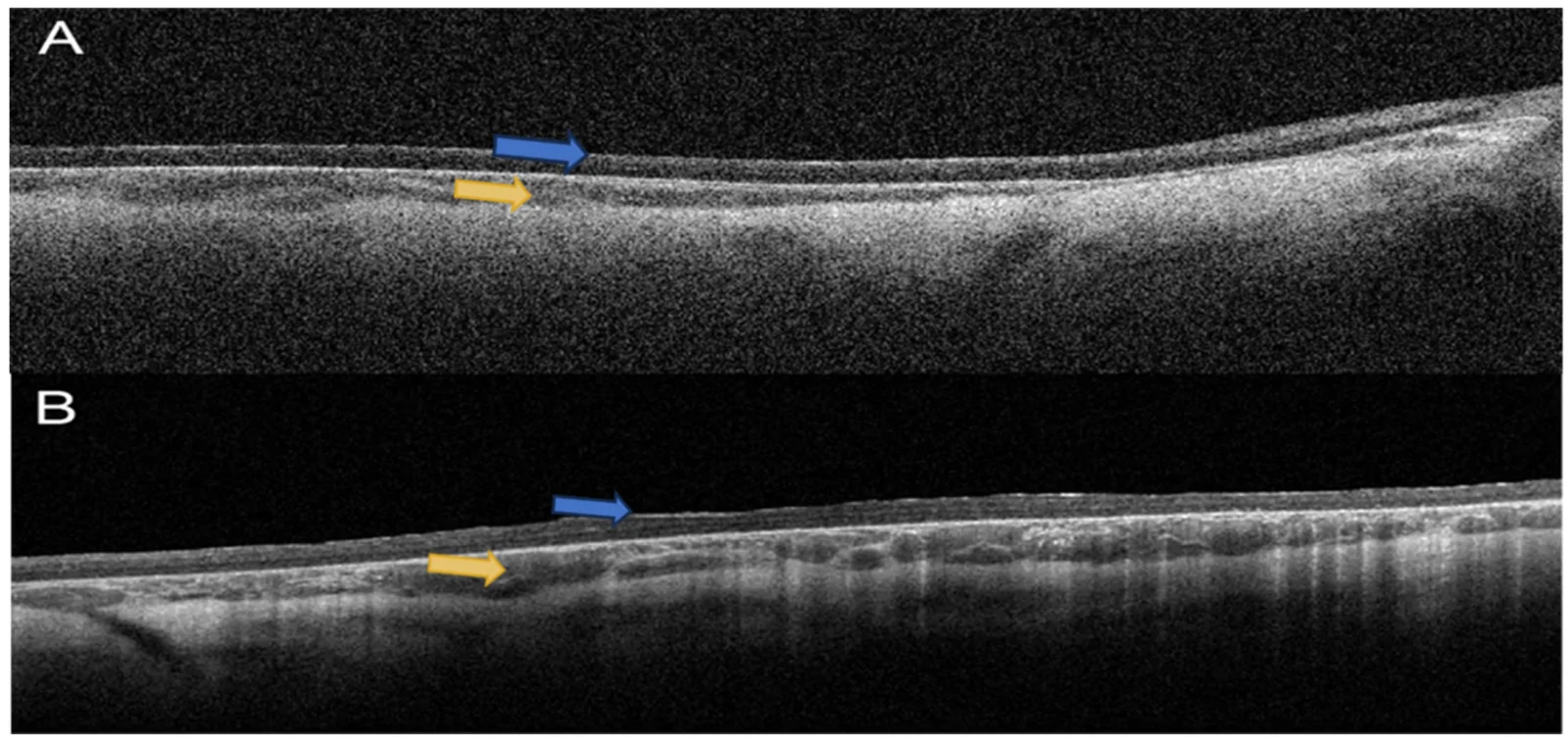
Patient 3. Heidelberg wide-field optical coherence tomography of the macula of the right (**A**) and left (**B**) eye showing severe myopic macular atrophy (blue arrows) and choroidal thinning (yellow arrows).

A 6-year-old female was referred for evaluation of nystagmus and macular changes, previously misdiagnosed with ocular albinism at 6 months of age. On examination at age 6, she was found to have high myopia (−16.00 D OD, −15.00 D OS) and BCVA of counting fingers OU. Fundus examination demonstrated prominent macular atrophy and vitreous degeneration bilaterally (Figure 7). SD-OCT revealed extensive outer retinal atrophy at the macula (Figure 8). Neuroimaging confirmed the presence of an occipital encephalocele, representing the most severe end of the KS occipital phenotypic spectrum.

NGS panel testing (Invitae 330-gene IRD panel) identified two heterozygous variants in *COL18A1*: a frameshift variant c.755del p.Gly252Alafs*19, classified as pathogenic by the testing laboratory per ACMG/AMP criteria [10], and a variant of uncertain significance (VUS) c.3827C > T p.Ser1276Leu. Parental segregation was performed with the NGS panel test. The pathogenic frameshift variant c.755del p.Gly252Alafs*19 was detected in the mother, and the variant of uncertain significance c.3827C > T p.Ser1276Leu was detected in the father. The c.3827C > T p.Ser1276Leu variant has not been previously reported in the literature and is classified as a VUS pending further functional and segregation data. Confirmation that this VUS and the pathogenic c.755del p.Gly252Alafs*19 variant are in trans establishes the compound heterozygous genotype but does not, by itself, establish the pathogenicity of the VUS; given the consistent KS phenotype in this patient, we consider this a candidate molecular diagnosis pending further functional and classification data.

This patient’s younger sibling presented at 7 months of age with a refraction of +1.00 OU, large-angle exotropia, and a large macula off retinal detachment with proliferative vitreoretinopathy of the right eye. Knobloch syndrome is suspected, but he has yet to receive genetic testing at the time of this article.

### 3.4. Patient 4

**Figure 9:**
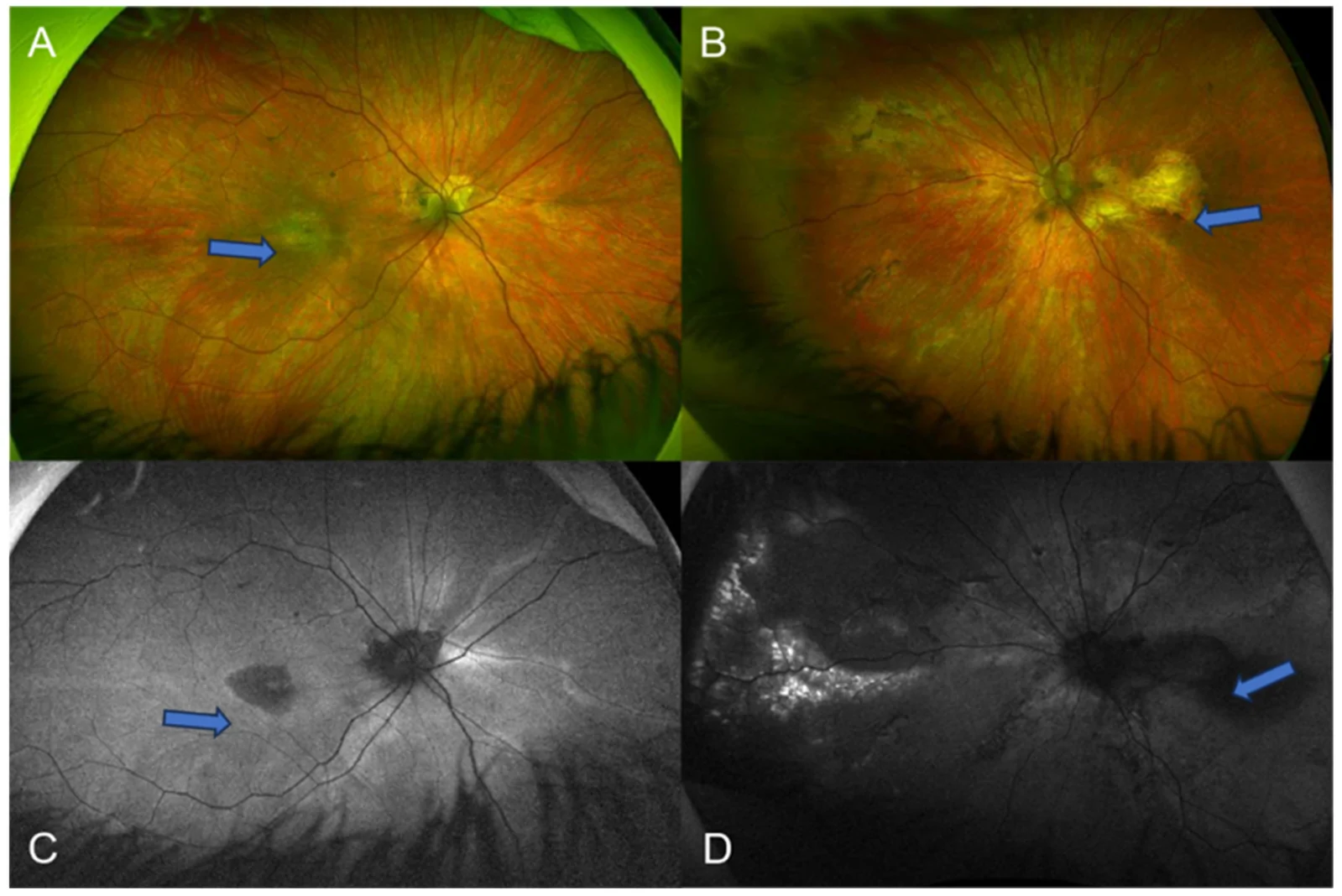
Patient 4. Optos ultra-widefield color fundus photos (**A**,**B**) and fundus autofluorescence photos (**C**,**D**) of the right (**A**,**C**) and left (**B**,**D**) eye showing severe myopic macular atrophy and reduced autofluorescence (blue arrows).

**Figure 10:**
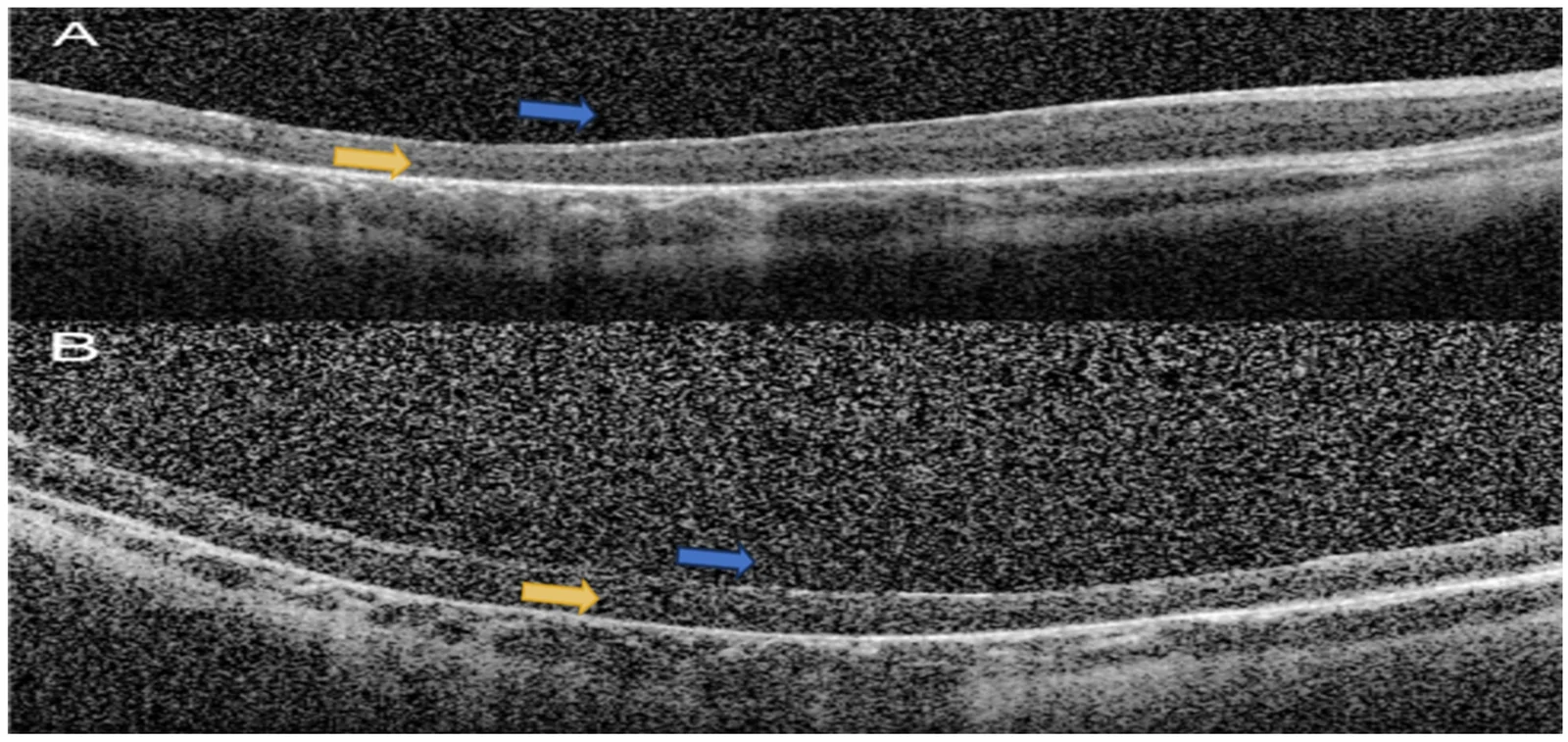
Patient 4. Heidelberg wide-field optical coherence tomography of the macula of the right (**A**) and left (**B**) eye showing severe myopic macular atrophy (blue arrows) and choroidal thinning (yellow arrows).

An 8-year-old male was referred at 11 months of age with parental concern for amblyopia. At age 11 months, examination revealed myopia (−4.50 D OD, −5.25 OS), exotropia of 10 prism diopters, and visual acuity of 20/190 by Teller acuity cards. By age 8 years, his refraction was −11.00 OU with BCVA of 20/125 OD and 20/400 OS. Fundus examination demonstrated macular atrophy and vitreous degeneration bilaterally (Figure 9). SD-OCT confirmed extensive macular outer retinal atrophy bilaterally (Figure 10).

**Figure 11:**
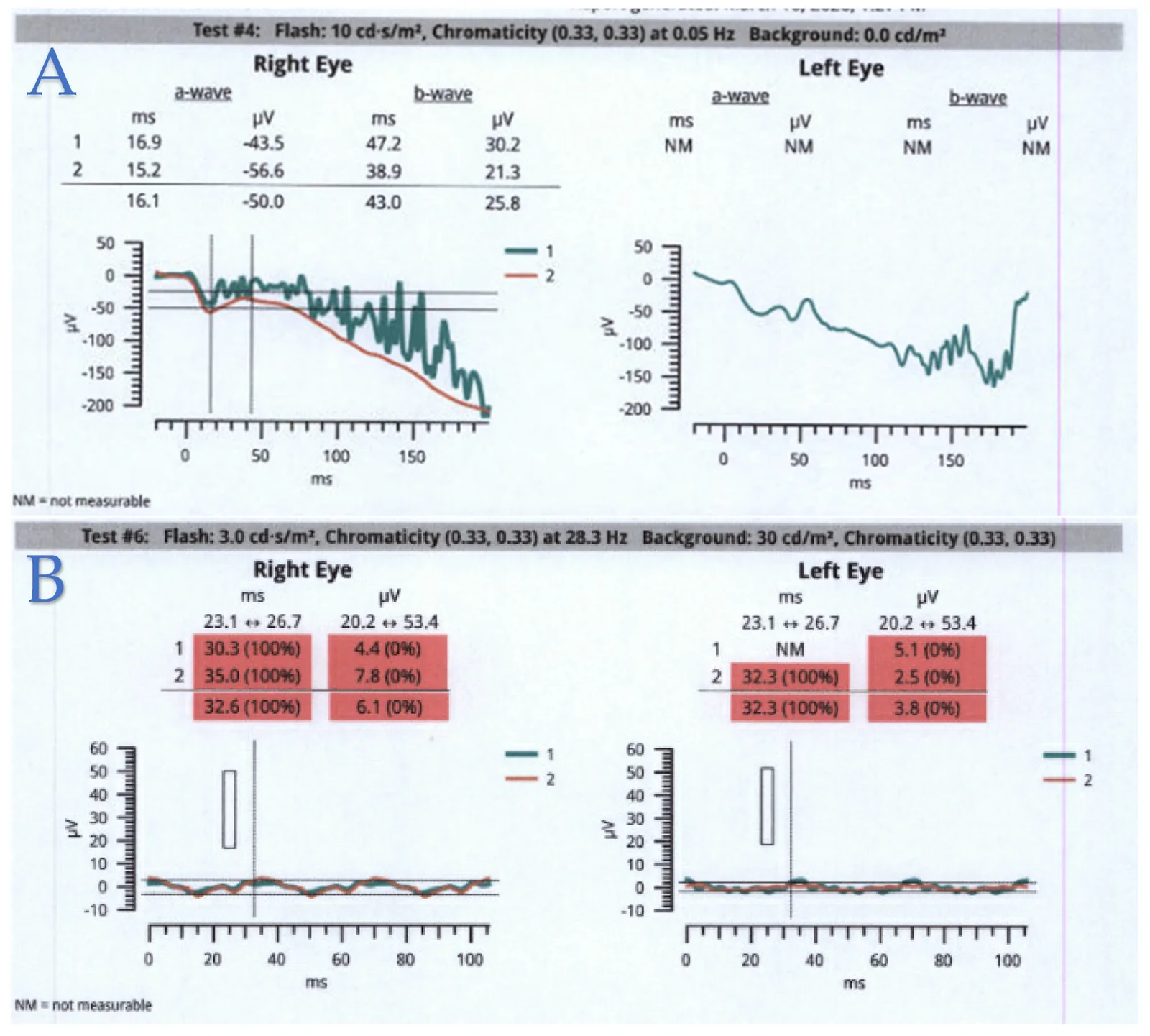
Patient 4 at age 4. (**A**) RETeval dark adapted 10 cd·s/m^2^ recordings of the right eye and left eye indicating a negative waveform. (**B**) Light-adapted (LA) 28.3 Hz 3.0 cd·s/m^2^ flicker recording of the right eye and the left eye showing a residual and delayed response for the right and left eye. The single-flash averaged responses for LA conditions 3.0 cd·s/m^2^ DA 0.01 cd·s/m^2^ and 3.00 cd·s/m^2^ were not measurable (NM) in one or both eyes.

At age 1, ERGs were recorded during EUA using a RETeval modified protocol. We tested the photopic responses in the right eye at 3.0 cd·s/m^2^ while the left eye was patched for dark adaptation. The responses were barely recordable and delayed for the photopic flicker stimulus 28.3 Hz 3.0 cd·s/m^2^ in the right eye and appeared electronegative for the 3.0 cd·s/m^2^ stimulus for the left eye. At age 4, the RETeval ERG was repeated in clinic using a modified ISCEV protocol (ISCEV6 Step Dark First cd·s/m^2^) and appeared electronegative for the 10 cd·s/m^2^ stimulus (Figure 11). Systemic evaluation was notable for global developmental delay. Chart review indicates that fetal ultrasound in utero showed concern for hydronephrosis. Repeat kidney ultrasound at 1 year of age showed normal kidneys and laboratory testing indicated normal kidney function. No occipital scalp defect or encephalocele was identified on neuroimaging.

WES (GeneDx) identified a novel heterozygous frameshift variant, c.2673dupC p.Gly892Argfs*9, and a pathogenic variant, c.3514_3515del p.Leu1172Valfs*72, in *COL18A1*. The variant c.2673dupC p.Gly892Argfs*9 has not been previously described in the literature and was classified as likely pathogenic by the testing laboratory per ACMG/AMP criteria, consistent with its frameshift mechanism and predicted loss-of-function effect [10]. Parental segregation testing could not be completed due to social constraints.

## 4. Discussion

We report four patients with clinical features of Knobloch syndrome and pathogenic, likely pathogenic, or candidate *COL18A1* variants identified from a 627-patient inherited retinal disease cohort at a single academic institution, representing 0.64% of IRD patients seen at WVU during the study period. This figure should not be interpreted as a population-based prevalence estimate, given the referral and testing bias inherent to a single-center cohort. All four patients demonstrated the consistent core ophthalmic phenotype of KS: nystagmus, high myopia, macular atrophy, vitreous degeneration, and severely abnormal ERG findings where testing was performed. This is consistent with prior reports highlighting these ophthalmic features of KS as a reliable diagnostic triad [4,5,6]. This paper expands upon a previously published abstract presented at the Association for Research in Vision and Ophthalmology [14].

High myopia is a defining and universal feature of KS, reflecting the structural role of type XVIII collagen in vitreoretinal development and the anchoring of vitreous fibrils to the inner limiting membrane [3]. The degree of myopia in our cohort ranged from −9.00 D to −19.00 D, consistent with prior reports. Khan et al. described high myopia ranging from −10 to −20 diopters in a pediatric Saudi cohort, noting that the ophthalmic phenotype alone is sufficient to guide clinical diagnosis in experienced hands [4]. Hull et al. similarly found high myopia ranging from −10 D to −35 D in at least one eye in a majority of patients in their multi-center cohort, emphasizing that ophthalmic features precede and often dominate the clinical presentation [5].

Macular atrophy and vitreous degeneration were present in all four of our patients, consistent with prior series [5,8,15]. These findings reflect the basement membrane dysfunction caused by loss of collagen XVIII and its C-terminal fragment endostatin, which regulates ocular angiogenesis, vitreous structure, and retinal pigment epithelial function [3]. Levinger et al. reported macular atrophy and coloboma in four Israeli children with KS, demonstrating that macular structural abnormalities are a consistent finding irrespective of specific variant [8]. The Goldmann visual field examinations in our patient revealed markedly constricted fields reflecting the severity of peripheral retinal involvement.

ERGs were obtained for Patients 1 and 4. Patient 1 demonstrated reduced amplitudes and prolonged implicit times across all conditions at age 11, and Patient 4 had a barely recordable ERG at age 1 and an electronegative-appearing ERG at age 4 years. This is consistent with reports from Hull et al. and Levinger et al. demonstrating a cone–rod pattern of dysfunction in KS and confirms that KS can result in profound, early onset retinal dysfunction detectable by electrodiagnostic testing [5,8].

The systemic phenotype in our cohort demonstrated substantial variability, consistent with the known heterogeneity of KS [7,9]. Patient 3 was the only patient to present with frank occipital encephalocele, the historically defining systemic feature of KS. Patient 1 presented with vertex scalp lesions consistent with aplasia cutis congenita—a recognized, though non-encephalocele, midline finding in KS [13]. Seaver et al. redefined the syndromic occipital features of KS to include scalp defects without frank encephalocele, which aligns with Patient 1’s presentation [13]. Patient 2 had a renal structural anomaly. Patient 4 had antenatal hydronephrosis suspected on fetal ultrasound, which resolved on repeat postnatal imaging, as well as global developmental delay. Williams et al. previously reported renal anomalies as a phenotypic variant in KS, indicating these findings represent part of the broader systemic spectrum [6]. Caglayan et al. further documented central nervous system malformations and seizures as occasional KS features, consistent with Patient 1’s seizure disorder [9].

Regarding genotype, four distinct *COL18A1* variants were identified in our cohort. The recurrent homozygous frameshift variant c.3514_3515del p.Leu1172Valfs*72 was identified in both Patients 1 and 2 from different families. This variant has been reported in multiple prior unrelated KS families, suggesting it may represent a recurrent variant in certain populations [5]. Patient 3 was found to be compound heterozygous for the pathogenic frameshift c.755del p.Gly252Alafs*19 and a novel VUS, c.3827C > T p.Ser1276Leu. This missense variant affects a conserved serine residue within the C-terminal domain of type XVIII collagen. While the precise functional impact of this substitution has not yet been established, parental segregation testing performed by NGS panel confirmed that c.755del p.Gly252Alafs*19 was maternally inherited and c.3827C > T p.Ser1276Leu was paternally inherited, establishing a trans configuration between the two variants. This segregation finding establishes a trans configuration between the two variants but does not, by itself, establish the pathogenicity of c.3827C > T p.Ser1276Leu; the co-occurrence of this VUS in trans with a confirmed pathogenic allele in a patient with a classic KS phenotype supports its consideration as a candidate molecular diagnosis. Functional studies are needed to clarify the classification of this variant.

The second novel variant, c.2673dupC p.Gly892Argfs*9 (Patient 4), represents a single-base duplication predicted to cause a frameshift and premature termination within the central collagenous domain of type XVIII collagen. Loss-of-function frameshift variants are the predominant mutational mechanism in KS, consistent with the autosomal recessive inheritance pattern and the critical structural role of type XVIII collagen [2,5]. In Patient 4, this variant was identified in the heterozygous state together with the recurrent pathogenic variant c.3514_3515del p.Leu1172Valfs*72, the same variant identified in Patients 1 and 2. Parental samples were not submitted for testing, so the phase (cis versus trans) of these two variants could not be determined from sequencing data alone; Patient 4 is therefore best described as harboring two heterozygous*COL18A1* variants of undetermined phase, rather than a confirmed compound heterozygous genotype, pending parental segregation testing.

This series has several limitations inherent to its retrospective design and the rarity of KS. The small sample size precludes firm genotype–phenotype conclusions. Longitudinal follow-up data, including detailed retinal imaging at standardized time points, are lacking for several patients. Functional studies of the two previously unreported variants have not been performed. As a single-center cohort drawn from patients referred for inherited retinal disease evaluation, this series is also subject to referral and testing bias, which limits its generalizability. *COL18A1* was not included on all genetic testing platforms and panel versions used across the broader cohort, particularly prior to 2020; because a systematic re-review of all 627 patients’ testing results was not feasible, it is possible that additional patients with KS-related phenotypes were not identified because of this non-uniform gene coverage. Despite these limitations, this series contributes to the growing literature on *COL18A1*-associated Knobloch syndrome by expanding the repertoire of documented and candidate *COL18A1* pathogenic variants and characterizing the ophthalmic phenotype with multimodal imaging including OCT, fundus photos, and Goldmann kinetic perimetry.

## 5. Conclusions

We report four patients with clinical features of Knobloch syndrome and pathogenic, likely pathogenic, or candidate *COL18A1* variants, including two previously unreported *COL18A1* variants: the frameshift variant c.2673dupC p.Gly892Argfs*9 (likely pathogenic) and the missense variant c.3827C > T p.Ser1276Leu (VUS). Our series reaffirms the consistency of the core ophthalmic phenotype of KS—nystagmus, high myopia, macular atrophy, vitreous degeneration, and severely abnormal ERG—while demonstrating significant variability in systemic manifestations. KS should be considered in the differential diagnosis of any infant or child presenting with early onset nystagmus and high myopia, particularly in the setting of macular atrophy and profoundly abnormal ERG findings. Genetic testing with a comprehensive inherited retinal disease panel or WES is essential for accurate diagnosis and genetic counseling. Identification of previously unreported variants in this rare syndrome contributes to the expanding understanding of *COL18A1*-related disease.

## Figures and Tables

**Table 1:** Clinical and genetic summary of four patients with Knobloch syndrome.

Patient/Age/Sex	*COL18A1* Variant(s)	BCVA	Refraction	Ophthalmic Findings	Systemic Findings
1/31F	c.3514_3515del p.Leu1172Valfs*72 (homozygous, pathogenic)	20/400 OU (aphakic contacts)	−13.00 OD −14.00 OS	Posterior staphyloma; bilateral subluxed lenses with cataract (s/p vitrectomy and cataract extraction); macular atrophy; reduced-amplitude ERG; constricted Goldmann VF	Seizure disorder; aplasia cutis congenita (vertex scalp)
2/6F	c.3514_3515del p.Leu1172Valfs*72 (homozygous, pathogenic)	20/250 OU	−9.00 OD −19.00 OS	Nystagmus; exotropia; optic nerve hypoplasia, anisomyopia; macular atrophy; vitreous degeneration	Renal anomaly
3/6F	c.755del p.Gly252Alafs*19 (pathogenic) c.3827C > T p.Ser1276Leu (VUS) (compound heterozygous)	CF OU	−16.00 OD −15.00 OS	Nystagmus; macular atrophy; vitreous degeneration; constricted Goldmann VF	Occipital encephalocele
4/8M	c.3514_3515del p.Leu1172Valfs*72 (pathogenic) c.2673dupC p.Gly892Argfs*9 (heterozygous, likely pathogenic)	20/125 OD 20/400 OS	−11.00 OU	Nystagmus; exotropia; macular atrophy; vitreous degeneration; severely abnormal ERG (scotopic and photopic); constricted Goldmann VF	Global developmental delay; antenatal hydronephrosis (resolved)

BCVA = best-corrected visual acuity; CF = count fingers; ERG = electroretinogram; VF = visual field; VUS = variant of uncertain significance; s/p = status post.

**Table 2:** Confirmed variant-level annotation for identified *COL18A1* variants.

**Variant (cDNA/Protein)**	**Reference Transcript (Exon)**	**Genomic Coordinate** **(GRCh38)**	**Genomic Coordinate** **(GRCh37)**	**Zygosity**	**Inheritance/Phase**
c.3514_3515del p.Leu1172Valfs*72 (Pts 1, 2, 4)	NM_130445.4 (Exon 41)	g.45510091_45510092del (chr21)	g.46930005_46930006del (chr21)	Homozygous (Pts 1, 2) Heterozygous (Pt 4)	N/A (Pts 1, 2) Not determined (Pt 4)
c.755del p.Gly252Alafs*19 (Pt 3)	NM_130445.4 (Exon 5)	g.45475492del (chr21)	g.46895406del (chr21)	Heterozygous	Maternal; in trans with c.3827C > T (paternal)
c.3827C > T p.Ser1276Leu (Pt 3)	NM_130445.4 (Exon 42)	g.45512214C > T (chr21)	g.46932128C > T (chr21)	Heterozygous	Paternal; in trans with c.755del (maternal)
c.2673dupC p.Gly892Argfs*9 (Pt 4)	NM_130445.4 (Exon 32)	g.45497651dup (chr21)	g.46917565dup (chr21)	Heterozygous	Not determined
**Variant (cDNA/Protein)**	**gnomAD Frequency**	**ClinVar Status**	**Predicted Consequence**	**Classification**	**Classification Date**
c.3514_3515del p.Leu1172Valfs*72 (Pts 1, 2, 4)	0.030% (rs398122391); no homozygotes reported	Pathogenic (VCV000065410)	Frameshift → premature stop; NMD substrate	Pathogenic	Pt 1 (Invitae): 6 June 2023 Pt 2 (GeneDx): 15 March 2022 Pt 4 (GeneDx): 2 October 2020
c.755del p.Gly252Alafs*19 (Pt 3)	Not present in population databases	No entry	Frameshift → premature stop; NMD substrate	Pathogenic	22 January 2025 (Invitae)
c.3827C > T p.Ser1276Leu (Pt 3)	Present, but data quality flagged as unreliable at this position	No entry	Missense (Ser → Leu)	Variant of uncertain significance	22 January 2025 (Invitae)
c.2673dupC p.Gly892Argfs*9 (Pt 4)	0.0097% (17/175,050 alleles); no homozygotes reported	No entry	Frameshift → premature stop; NMD substrate	Likely pathogenic	2 October 2020 (GeneDx)

Abbreviations: gnomAD = Genome Aggregation Database; ClinVar = NCBI ClinVar database; LOVD = Leiden Open Variation Database; NMD = nonsense-mediated decay; PTC = premature termination codon; VUS = variant of uncertain significance; N/A = not applicable; Pt/Pts = patient(s). *Sources:* Reference transcripts and variant classifications are as reported by the originating clinical laboratory (Invitae Inherited Retinal Disorders panel: Patients 1 and 3; GeneDx whole-exome sequencing: Patients 2 and 4). Genomic coordinates (GRCh38 and GRCh37) were independently confirmed using VariantValidator (variantvalidator.org) against each variant’s laboratory-reported transcript. Population frequency, zygosity, and ClinVar status are as reported by the originating laboratory. Phase for Patient 3’s variants was confirmed by parental panel testing (maternal origin for c.755del, paternal origin for c.3827C > T). Phase for Patient 4’s variants was not determined, as parental samples were not submitted for analysis.

## Data Availability

The original contributions presented in this study are included in the article. Further inquiries can be directed to the corresponding author.
